# Genetic Diversity Trends in the Cultivated Potato: A Spatiotemporal Overview

**DOI:** 10.3390/biology11040604

**Published:** 2022-04-15

**Authors:** Martin Spanoghe, Thierry Marique, Alexandra Nirsha, Florence Esnault, Deborah Lanterbecq

**Affiliations:** 1 Laboratoire de Biotechnologie et Biologie Appliquée, Haute Ecole Provinciale de Hainaut-CONDORCET, Digue de Cuesmes 29, 7000 Mons, Belgium; thierry.marique@condorcet.be (T.M.); deborah.lanterbecq@condorcet.be (D.L.); 2 Centre Pour l’Agronomie et l’Agro-Industrie de la Province du Hainaut (CARAH), Paul Pastur 11, 7800 Ath, Belgium; a.nirsha@carah.be; 3 Institut de Génétique Environnement et Protection des Plantes (IGEPP), INRAE, Institut Agro, Université Rennes 1, 29260 Ploudaniel, France; florence.esnault@inrae.fr; 4 Hainaut Analyses (HA), Bd Sainctelette 55, 7000 Mons, Belgium

**Keywords:** genetic diversity, self-organizing map, SSR, potato breeding, *Solanum tuberosum*, sustainable crop production

## Abstract

**Simple Summary:**

Monitoring the change in genetic diversity over time and space in crop species is essential to facilitating further improvement. As the world’s most important tuber crop for human consumption, and an ideal candidate to help address global food security, the cultivated potato deserves in-depth study in this regard. In this overview, some aspects of spatiotemporal diversity assessment in the cultivated potato are examined with the aim of promoting appropriate strategies for breeding programs in line with challenges relating to sustainable crop production.

**Abstract:**

We investigated the changes in genetic diversity over time and space of the cultivated potato (*Solanum tuberosum* L.) for the period pre-1800 to 2021. A substantial panel of 1219 potato varieties, belonging to different spatiotemporal groups, was examined using a set of 35 microsatellite markers (SSR). Genotypic data covering a total of 407 alleles was analyzed using both self-organizing map (SOM) and discriminant analysis of principal components (DAPC) de novo and a priori clustering methods, respectively. Data analysis based on different models of genetic structuring provided evidence of (1) at least two early lineages that have been maintained since their initial introduction from the Andes into Europe in the 16th century, followed by later ones coming from reintroduction events from the US in the mid-1800s; (2) a level of diversity that has gradually evolved throughout the studied time periods and areas, with the most modern variety groups encompassing most of the diversity found in earlier decades; (3) the emergence of new genetic groups within the current population due to increases in the use of germplasm enhancement practices using exotic germplasms. In addition, analysis revealed significant genetic differentiation both among and within the spatiotemporal groups of germplasm studied. Our results therefore highlight that no major genetic narrowing events have occurred within the cultivated potato over the past three centuries. On the contrary, the genetic base shows promising signs of improvement, thanks to extensive breeding work that is gaining momentum. This overview could be drawn on not only to understand better how past decisions have impacted the current genetic cultivated potato resources, but also to develop appropriate new strategies for breeding programs consistent with the socio-economic and sustainability challenges faced by agrifood systems.

## 1. Introduction

Crop breeding plays a crucial role in the production of high-quality food and, therefore, in improving global food security. In this regard, increasing genetic diversity is essential to provide opportunities for further improvement of crop species and to maintain agroecosystem functioning [1]. To achieve sustainable crop production in the face of future challenges such as climate change, it is also of major interest to monitor fluctuations in the genetic diversity of crops over time, caused by both biotic and abiotic constraints and by anthropogenic breeding practices [2,3]. As the world’s most important tuber crop, and the third most important crop grown for human consumption, the cultivated potato is a prime candidate crop for helping address food security worldwide [4]. This is due to its highly diverse distribution, the extent of current cultivation and demand, and the ease of its culinary use, especially in developing countries with high levels of poverty, hunger, and malnutrition. For these reasons, it deserves thorough study.

The assessment of the trends in a crop’s genetic diversity over time and space can be addressed through a number of approaches, which depend on the different views and interpretations that exist of the concept itself [2]. In this study, three interconnected and complementary approaches, based on molecular marker data, were used to assess spatiotemporal trends in cultivated potatoes.

The first traces the evolution of the cultivated potato from its native to its modern form, particularly in terms of how introductions and subsequent past decisions have shaped modern lineages. Information on the parental origin of potato varieties that have been cultivated, or themselves used as parental lines for further selection, provides a wealth of information on historical breeding choices [5]. Such historical records allow a better understanding of the current population structure and can therefore provide relevant data for breeders to use when making the right decisions in their respective breeding programs [6,7]. As demonstrated in the modern tetraploid potato crop, information about pedigree is essential to achieving suitable association mapping [8,9], for deciphering genome-wide conserved patterns in elite potato parental lines [10], and for studies on the inheritance of extreme resistance [11]. The use of genealogical information is also helpful in estimating breeding values and improving genetic gains for low heritability traits in potato breeding [12,13]. Although many studies have attempted to identify the origins of the cultivated potato in Europe [14,15,16,17,18,19,20], and how much it has diversified [6,21], many questions remain. In particular, the lack of information on the genetic background of many varieties prevents precise tracing of how the lineages in question were established and where, following several distinct introduction events and subsequent diversification [10,22]. However, such ‘orphan cultivars’ could provide unsuspected genetic resources that could be exploited in breeding programs.

A second approach assesses a possible reduction of potato genetic diversity caused either by modern agricultural practices (or by fortuitous decisions in the past) or by environmental changes. The concept of genetic erosion relies on the loss of alleles that severely affect the genetic vulnerability of crops to biotic and abiotic stress [23]. The impact of such risks has been well documented, for example the occurrence of epidemics such as the Irish potato blight in the 1840s, the great French wine blight in the late 1860s, and the U.S.A. corn blight in the 1970s. Quantifying the occurrence of such events in relation to the crop being studied is therefore essential. Throughout its history, the cultivated potato has experienced severe and repeated selection pressures resulting in some sequential putative genetic bottleneck events, including domestication (6000 BCE), adaptation to a long photoperiod (pre-Colombian) and to temperate regions (1450–1900), late blight pandemics (mid-1900s), seed tuber-borne viruses (persistent), and trade barriers (19th–20th century) [24]. Many potato genotypes have also been lost due to problems of tuber propagation, including virus accumulation, seed storage losses, and loss of fertility, which have led to an increase in the narrowness of its genetic base [25,26]. However, the few studies addressing this issue have shown that genetic erosion is not observed in the cultivated potato [27,28]. Nevertheless, the genetic composition of modern cultivars has been proven to essentially result from the contribution of major founders [28,29].

A third approach measures the effectiveness of the use of exotic germplasm in modern selection, particularly that of wild crop relatives and landraces. Such breeding practices are key means of restoring genetic diversity that has been lost over time as result of domestication, migration, disease, and other causes of genetic bottlenecks [24]. This allows untapped adaptive potential, such as enhanced disease resistance and quality traits that can be targeted for the improvement of existing cultivated crop varieties [21,30]. Several approaches exist for the use of exotic genetic resources in germplasm improvement, including incorporation (e.g., base broadening by analytical reproduction), introgression (e.g., backcrossing) and genetic manipulation (e.g., transgenesis) [31,32]. In a recent inventory of crop wild relatives (CWR) that could help strengthen global food security, the potato achieved the highest CWR diversity score [33]. In the potato, the use in selection of Andean cultivated landraces of the groups Phureja, Andigena, and Tuberosum, as well as many wild species, has taken place since the 1890s [21,34] and resulted in the release of a multitude of improved varieties. For example, resistance to late blight was mostly incorporated into potato cultivars from *S. demissum* [35], resistance to cyst nematodes from group Andigena [36], but also from *S. vernei* and *S. spegazzini* [37], resistance to virus X from *S. acaule,* and to virus Y from *S. stoloniferum* [25]. Other wild species have conferred resistance to a variety of viral, fungal, and bacterial diseases, as well as insect pests of potatoes [38]. Agronomic traits such as yield, specific gravity, chipping quality, and suppression of enzymatic browning have also been improved using wild species [31]. Although these species possess great variability in many desirable agronomic characters, that diversity reservoir seems to be largely unexplored, meaning that the cultivated potato’s genetic base appears to remain narrow [39,40]. It also appears that introgression from only a small proportion of wild potato relatives is found in potato cultivars [35]. Nevertheless, the recent detection of genomic admixture signals in modern European potatoes has been attributed to breeding using wild Solanum species [20]. Hence, it would be of interest to quantify the real impact of these practices on current global germplasm from an exhaustive study of spatiotemporal genotypic data.

To adequately address these three approaches to evaluating the fluctuations in the spatiotemporal genetic diversity of the cultivated potato, we applied two clustering methods, self-organizing map (SOM) [41] and discriminant analysis of principal components (DAPC) [42], over a substantial panel of potato lines using 35 informative microsatellite markers (SSR). SOM is applied with no prior knowledge of data organization, meaning that any cluster detection is performed de novo, whereas DAPC is applied using prior knowledge of data organization, meaning that spatiotemporal groups are specified a priori. In addition, AMOVA analyses were used as a complementary multivariate tool to assess any genetic differentiation occurring among and between defined spatiotemporal groups of germplasm. To the best of our knowledge, this study is the first attempt to use these clustering methods, in combination with a wide range of both varieties and SSR markers, to examine the trends in genetic diversity over time and in space in the cultivated potato.

## 2. Materials and Methods

### 2.1. Plant Materials

The potato germplasm used in this study consisted of 1219 potato accessions, originating from Europe (1100), North America (70), South America (12), Asia (27), Africa (8), and Oceania (2). The plant material, released between pre-1800 and 2021, was obtained from several European breeder and maintainer collections as well as gene banks (see acknowledgments). Both the date of release and the geographical origins of each variety were registered in accordance with breeder information. For cultivars whose origin and/or date of release was uncertain, first cited appearance or description from historical documents and literature was registered as such. The panel was set up to cover as equally as possible each year during the period studied. The potato germplasm comprised 30 heirloom varieties (released before 1850), 205 old cultivars (released between 1850 and 1950) and 984 modern cultivars (released after 1950) (Table 1). The panel also included 9 tetraploid Andean accessions of *S. tuberosum* L. and 3 accessions of native potato from Chiloe Island, all used as references from the study of Esnault et al. 2014 [43], as well as some long-day-adapted selections of diploid and tetraploid cultivated potato species from the Andes. Two accessions are known to be diploid while all other are believed to be tetraploid. To assess the trends in diversity over time, the varieties were classified into a series of 50-year groups based on the date of their first appearance on the market (Table 1).

The online pedigree database [22] was used both to determine the parents most used to breeding within the studied germplasm and to build the identifier dataset. Pedigree validation based on kinship testing, parental inference analysis, and synonymy identifying, was then carried out to validate the identifier dataset, as described in a previous work [44]. In addition, steps about specific concerns with homologues (see Acknowledgments) were taken to guarantee as much as possible the authenticity of potato accessions. Lastly, any genotypes that were the subject of doubt were discarded from the study. An overview of the accession’s background information was compiled as an identifier set and is available in the Supplementary Materials (Table S1).

### 2.2. DNA Isolation and SSR Genotyping

Genomic DNA isolation was performed for each potato variety from tubers, leaves, or in vitro plantlets using the QuickPick Plant DNA kit as per the manufacturer’s protocol (Bio-Nobile, Turku, Finland). DNA extracts were quantified using a ND-3300 NanoDrop spectrofluorimeter (Thermo Scientific; Waltham, MA, USA).

Thirty-five SSR markers were selected from the literature based on the following criteria: (i) homogeneous repartition on the chromosomes; (ii) optimal amplification and resolution; (iii) ability to detect high rates of polymorphism; (iv) adequacy of observed fragment sizes with those reported in selected studies [45,46,47,48,49,50,51]; and (vi) suitability to be used in a multiplexed PCR reaction. The 35 SSR loci were distributed among five multiplex sets of 7 SSR markers (Table 2). The 5’ end of the forward primer of each pair was labeled with a fluorescent dye (6-FAM, HEX, or NED dyes; Table 2). The PCR amplifications were carried out on a Biosystems™ SimpliAmp Thermal Cycler (Applied Biosystems, Waltham, MA, USA) using a Kapa2G Fast Multiplex Mix (Kapa Biosystems, Boston, MA, USA) in a total reaction volume of 25 μL containing 1× Kapa2G Fast Multiplex Mix, 0.1–0.2 μM of each primer (Thermo Fisher Scientific, Waltham, MA, USA), and 15 ng of template DNA. The PCR cycling parameters were as follows: initial denaturing step of 3 min at 95 °C, then 30 cycles of 15 s at 95 °C, 30 s at 55 °C (Ta), 40 s at 72 °C, followed by a hold step of 1 min at 72 °C for final extension. After amplification, 1 μL fraction of each multiplex PCR products was transferred into 14 μL HiDi (Applied Biosystems, Waltham, MA, USA) containing 2.5% of GeneScan™ 400HD™ dye Size Standard (Applied Biosystems, Waltham, MA, USA). The PCR products were run on a SeqStudio Genetic Analyzer System (Applied Biosystems, Waltham, MA, USA) following standard run-module parameters. Estimations of fragment lengths of the PCR products were determined using GeneMapper Software 6.0 (Applied Biosystems, Waltham, MA, USA) and used to build a multilocus genotyping dataset for all potato accessions. To generate a dataset of SSR allele counts, DNA fragments were scored as either 1 or 0 (i.e., for presence or absence) for each accession and for all 35 SSR loci, considering each fragment as a dominant allele. We used dominant loci to overcome the constraints of the specific locus dosage during the scoring of polymorphic fragments [46]. In our case, this approach seemed easier to implement than the one, for example, suggested by Esselink et al. (2004) [52], since it meant we no longer had to define any extra haplotype or extra information in the genotypic model when unambiguous evidence of null alleles was available. This SSR dataset, named ‘‘input data,’’ constituted the base frame of the clustering analyses realized in this study and is available in the Supplementary Materials (Table S2). All subsequent multivariate analyses were conducted in RStudio version 1.4.1717 [53] using R version 4.1.1 [54].

### 2.3. Data Analysis

#### 2.3.1. Allelic Diversity of SSR Loci

Genetic diversity of SSR loci was estimated using the number of detected SSR alleles (Na) and the polymorphic information content (PIC) according to the Nei’s statistic [55]. The frequencies of both rare (frequency below 0.01) and unique alleles were determined by manual counting, assuming that no allelic dosage was considered in this study. The degree of heterozygosity H1 occurring in the potato panel was estimated based on the total frequency of accession occurrence for which distinct levels of heterozygosity (duplex, triplex and quadriplex) were observed.

#### 2.3.2. SOM Analysis

The self-organizing map (SOM) is a type of artificial neural network commonly used to model complex nonlinear relationships of large multidimensional data and sort it into decipherable clusters, while preserving the original topology of input data and following unsupervised learning rules [41]. SOM analysis was applied using the R package Kohonen version 3.0.10 [56] from the entire population of the potato accessions (*n* = 1219) with no prior information on data organization. The probability density function of the input data was estimated by performing a nonlinear projection of the genetic data, here expressed as a vector in a 1219-dimensional space, onto a two-dimensional network. The two-dimensional network consisted of 81 (9 × 9) output neurons arranged on a hexagonal network onto which the accessions could be assigned. A hexagonal network was preferred because it does not favor horizontal or vertical directions [57]. Since there are no strict rules regarding the number *N* of output neurons necessary to cluster the accessions, the SOM was trained using different map sizes and we selected the those that fitted best, according to the global quality criterion of the result based on the topographic error [58]. Details of the method can be found in Spanoghe et al. 2020 [59]. A learning process was performed to pattern the input vectors according to the SOM learning rules [57] and consisted here of 5000 iterations. We used a parallel batch training (*pbatch*) mode and Manhattan-type distance functions. Manhattan was preferred over default Euclidean distance functions because it gave clearer clustering patterns (greater number of empty neurons), although both methods led to similar outcomes.

#### 2.3.3. DAPC Analysis

Discriminant analysis of principal components (DAPC) is a multivariate method combining two analysis methods, discriminant analysis (DA) and principal component analysis (PCA), to assess population structure with the aim of maximizing among-group variation and minimizing within-group variation [42]. DAPC was conducted to infer the relationships of potato accessions within the entire population (*n* = 1219), using prior information on data organization. DAPC analyses were carried out using the R *adegenet* package version 2.1.4 [60]. Four a priori models of genetic structuring were examined as the following population identifiers: (1) germplasm of various continent origins, (2) germplasm of various country origins, (3) germplasm of three temporal groups (heirloom, old, modern) and (4) germplasm of 50-year breeding groups. For each DAPC analysis, a cross-validation function (*Xval.dapc*) was used to define the correct number of principal components (PCs) to be retained, being the lowest root mean square error according to R *adegenet* procedure [61]. Discriminant analysis (DA) was then conducted using *n* discriminant eigenvalues, where *n* = *number of a priori groups* − *1*. The resultant clusters were plotted in a scatterplot of the first and second linear discriminants (LD) of DAPC, with potato accessions as points and inertial ellipses around predefined groups.

#### 2.3.4. AMOVA Analysis

The analysis of molecular variance (AMOVA) [62] was performed for the same four models of genetic structuring using R package *pegas* 1.0–1 [63] to assess the partition of the total SSR variation into within and among-group variation components, but also to provide relative measures of inter-group genetic distance, in the form of the proportion of the remaining total SSR variation between any two groups (phi statistic; [62]). The significance of the resulting variance components and inter-group genetic distances was tested using 1000 random permutations.

## 3. Results

### 3.1. Allelic Diversity of SSR Loci

A total of 407 alleles were detected in the potato germplasm from the 35 SSR loci, of which 184 were rare alleles (45.2%) and 41 unique alleles (10.1%) (Table 2). The number of alleles per locus ranged from 6 (STM2005) to 22 (STI0023), with an average value of 11.6. The degree of heterozygosity H1 occurring in the potato germplasm was 0.893 and the mean of alleles per accession was 2.45. The PIC per SSR marker ranged from 0.346 (STG0006) to 0.834 (STI0036), with an average value of 0.734. The number, identity, and frequency of allele records that appeared and disappeared over time (50-year series) are available in the Supplementary Materials (Table S3).

### 3.2. SOM Analysis

**Figure 1:**
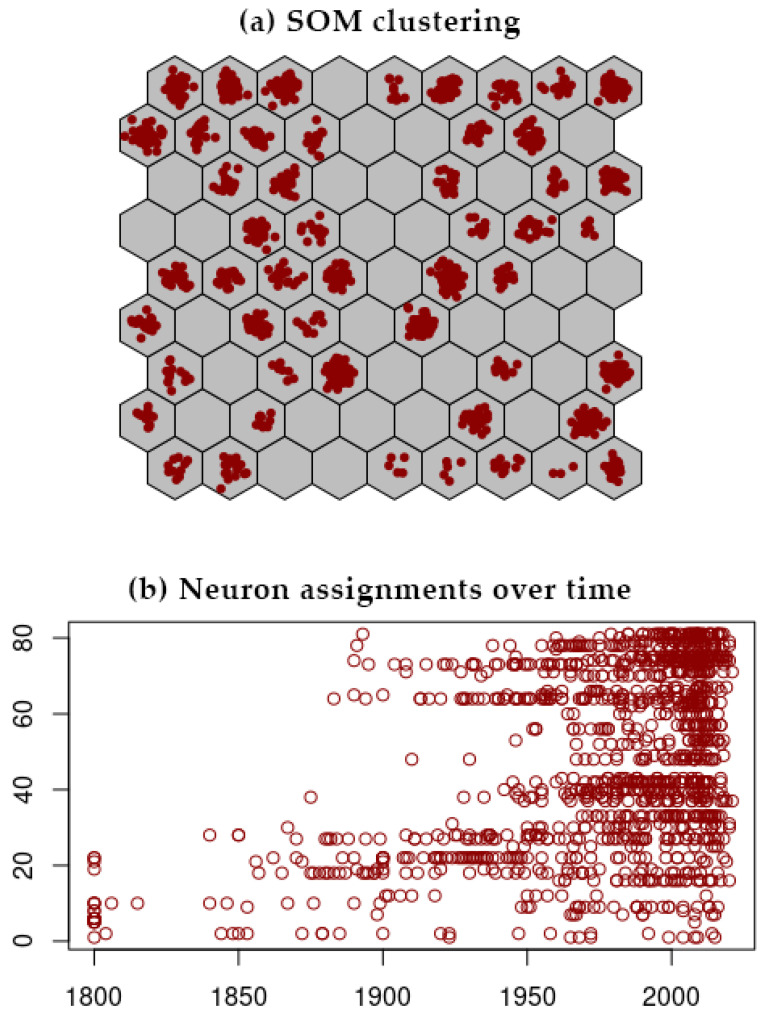
(**a**) SOM clustering for the germplasm of 1219 potato accessions of various origins. Each dot represents a potato accession. Neurons are numbered from bottom left of the grid (1) to top right (81); (**b**) Neuron assignments over time in years. Each circle represents a potato accession.

SOM analysis was processed using the complete dataset with no prior knowledge of data organization. During the learning process, SOM was trained with different assorted sizes of output layer, and the grid arranged into an output layer of hexagonal 9 × 9 neurons was selected as the optimal resolution for discerning plausible structured clusters (for larger dimensions, the assignment of any accession at some location in the SOM grid might no longer have been consistent with the topological preservation). As illustrated in Figure 1a, the SOM fitted the data within the multidimensional network into a network of clusters which were easily visualized. The most closely related potato accessions were grouped together in the same cluster, while genetically more distant accessions were located far away from each other.

One way to analyze the SOM clustering chronologically is to replot the neuron numbers for all accessions, as a function of time (Figure 1b). We can see potato accessions progressively fitting the neurons better over time, thus showing on which date a given neuron either starts or completes its own filling of accessions. The most ancient accessions (<1800) start at the bottom of the grid with a clear upwards progression through time. This is of particular interest since this pattern spontaneously appears without giving the SOM any information about chronology.

**Figure 2:**
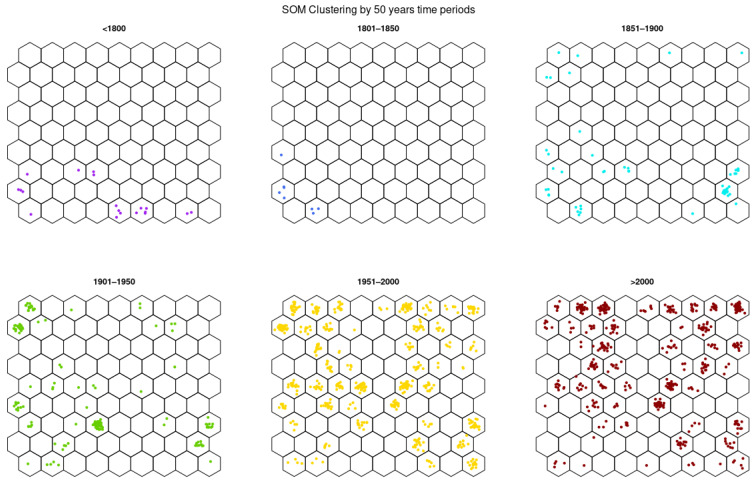
SOM clustering for the germplasm of 1219 potato accessions by 50-year breeding period ([<1800], [1801–1850], [1851–1900], [1901–1950], [1951–2000], [2000–2021]). Each dot represents a potato accession.

Figure 2 provides a more detailed plotting of chronology, displaying potato accessions in the SOM by 50-year period. Before 1800 (heirloom temporal group 1), four main neuron regions fitted by the oldest potato accessions are distinctly separated in space, indicating occurrence of four distinct genetic clusters from this period: Andigena native potatoes in neurons 5 and 6, *Vitelotte Noire* and *Johnny Gunter* in neuron 8, native potatoes from Chiloe Island in neurons 1 and 22, and heirloom varieties (i.e., *Makah Ozette*, *Native of the Canary Islands*, *Raudar Islenkar,* and *Yam*) in neurons 10 and 19. Between 1801 and 1850 (heirloom temporal group 2), potato accessions mainly remain around the previous heirloom cluster (neuron 10), implying that little genetic differentiation happens during this period. It should be noted that almost all other neurons have emptied. Between 1851 and 1900 (old temporal group 1), new clusters appear mainly bottom right and top left. This closely follows the major crises of potato late blight epidemics and corresponds to new introduction events. Between 1901 and 1950 (old temporal group 2), a reinforcement of preexisting clusters is found. From 1951 until 2021 (modern temporal groups 1 and 2), numerous new accessions appear both around preexisting clusters and in new regions of the SOM.

**Figure 3:**
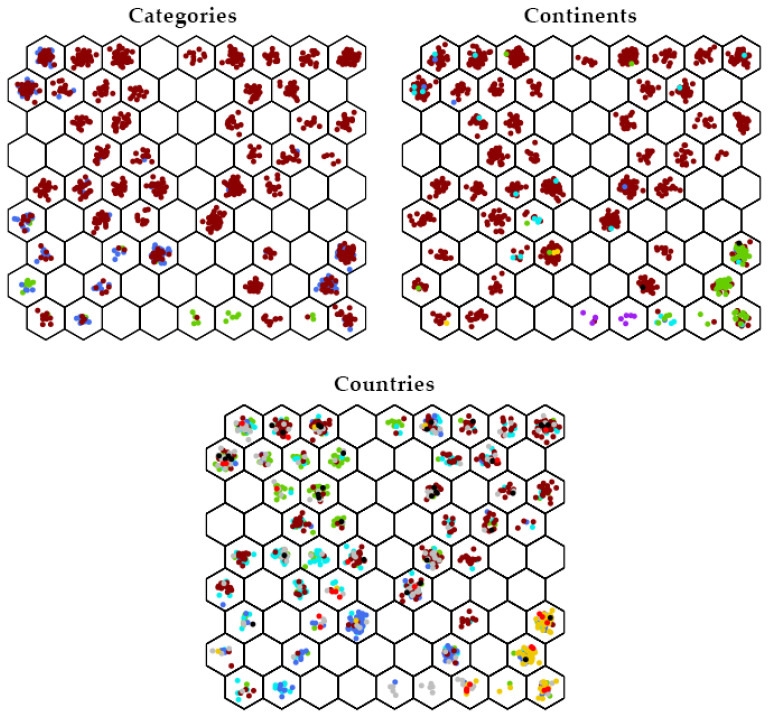
Distribution of potato accessions for 3 different sets of information. “Categories” shows heirloom accessions in green, old accessions in blue and modern accessions in red. “Continents” shows North American accessions in green, South American accessions in purple (Andigena) and in yellow (from Chiloe Island), European accessions in red, African accessions in blue, Asian accessions in cyan, Oceanian accessions in black. “Countries” shows accessions from Austria in black, from China in light red, from France in cyan, from Germany in green, from the Netherlands in dark red, from United Kingdom in blue, from the USA in yellow, and all other accessions in gray.

Figure 3 explores the SOM clustering of three other information sets. The set “Categories” shows the three chronological clusters’ progression from heirloom (green), to old (blue), to modern (red). As noted above, the oldest accessions start at the bottom of the grid with a clear upward progression through time. The set “Continents” clearly shows Andigena native accessions standing alone (neurons 5 and 6, purple). North American accessions in green and European accessions in red stand mostly apart, accessions from other continents being intermingled. The set “Countries” shows a more detailed geographical clustering, with, roughly clockwise: USA bottom right in yellow, UK mainly bottom in blue, France bottom left in cyan, Germany top left in green, China bottom right and top left in light red, Austria mainly top in black, and the Netherlands mainly top in dark red.

### 3.3. DAPC Analysis

Discriminant analysis of principal components (DAPC) was conducted to assess the relationships between potato accessions within the entire population (*n* = 1219) using four a priori spatiotemporal models of genetic structuring:

#### 3.3.1. Germplasm of Various Continental Origins

**Figure 4:**
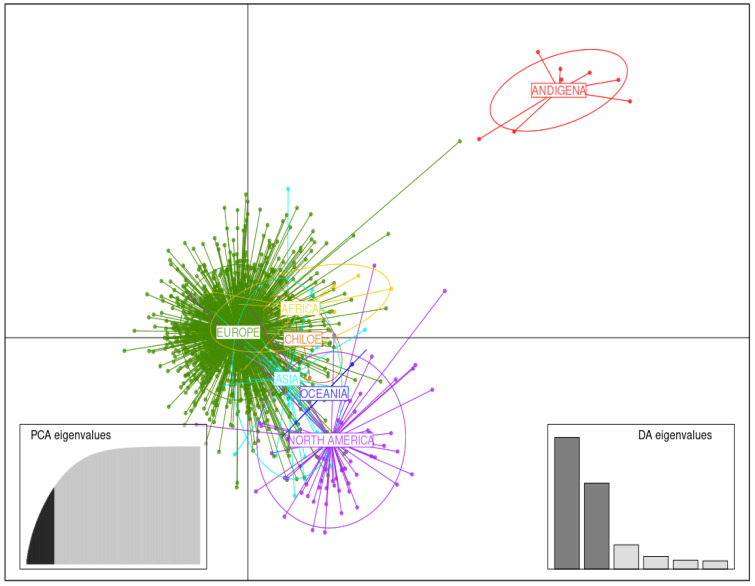
Discriminant analysis of principal components (DAPC) for the germplasm of 1219 potato accessions of various continental origins. The axes correspond to the first two linear discriminants (LD). Each ellipse represents a continent cluster (Africa, Asia, Europe, North America, Oceania, and South America (divided between Andigena accessions and accessions from Chiloe Island)) and each point represents an individual.

DAPC analysis using germplasm originating from various continents was carried out retaining 60 first PCs (about 65% of variance conserved) of PCA and six discriminant eigenvalues. Separation between continent clusters comprising potato accessions of Europe, North America, and South America (further divided between Andigena accessions and accessions from Chiloe Island) was visualized in the DAPC scatterplot with respect to the two first linear discriminants (LD) (Figure 4). In particular, the Andigena accessions subgroup is clearly set apart from all the other accessions, whereas the subgroup formed by the accessions from Chiloe Island is instead merged with them. The cluster comprising potato accessions from Africa appears to overlap strongly with the Europe cluster, the Oceania cluster with the North America cluster, and the Asia cluster with both. However, some potato accessions were found in a cluster other than that of their continent of origin. All these discrepancies are easily explained by close parental affiliations.

#### 3.3.2. Germplasm Originating in Various Countries

**Figure 5:**
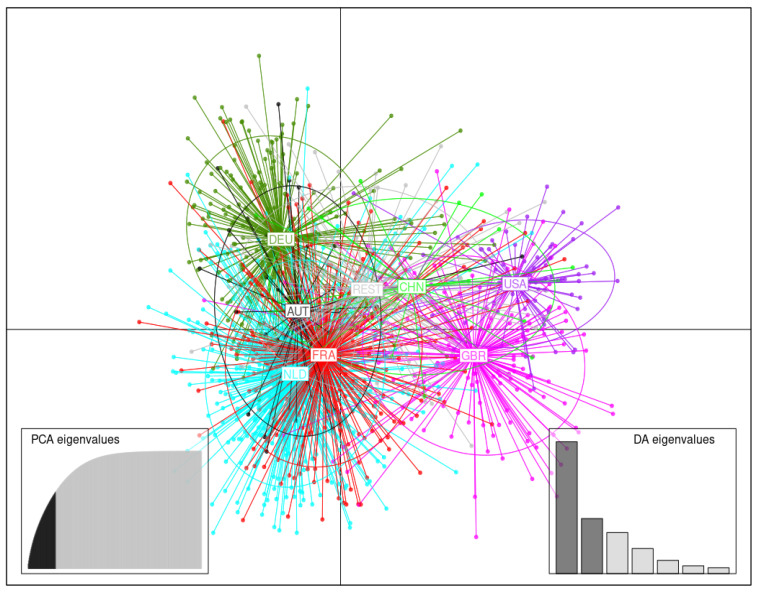
Discriminant analysis of principal components (DAPC) for the germplasm of 1219 potato accessions originating in various countries. The axes correspond to the first two linear discriminants (LD). Each ellipse represents a country cluster: Austria (AUT), China (CHN), France (FRA), Germany (DEU), the Netherlands (NLD), United Kingdom (GBR), United States (USA), and Rest (REST), and each point represents an individual.

DAPC analysis using germplasm originating in various countries was carried out retaining 60 first PCs (65% of variance conserved) of PCA and seven discriminant eigenvalues. In this model of structuring, only countries comprising more than 25 potato accessions were used to assess spatial interrelations, while the other countries were grouped into a single cluster called “Rest”. Although there is a partial overlap of the points and their resulting ellipses, the tendency of countries to be grouped according to their continent of origin is also found here (Figure 5). Thus, accessions from European countries are grouped together on the left of the DAPC scatterplot, those from the United Kingdom (GBR) at the bottom right, those from the United States (USA) on the middle right, and those from China are grouped between the American and European clusters, with respect to LD1 and LD2. Accessions included in the “Rest” cluster are found in the middle of the DAPC plot. Among the European accessions, accessions from Germany (DEU) are the most distant, whereas those from France (FRA) and the Netherlands (NLD) are closer to each other. Despite this rather clear spatial distribution, many potato accessions from one country are found in other clusters of origin.

#### 3.3.3. Germplasm of Three Temporal Groups

**Figure 6:**
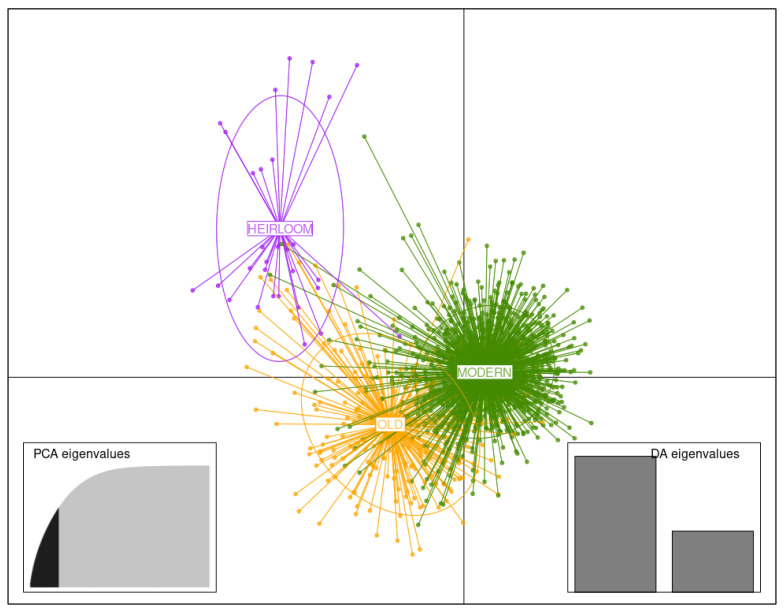
Discriminant analysis of principal components (DAPC) for the germplasm of 1219 potato accessions of three temporal groups. The axes correspond to the first two linear discriminants (LD). Each ellipse represents a temporal cluster (heirloom, old, modern), and each point represents an individual.

DAPC analysis using germplasm of three temporal groups (i.e., heirloom, old, and modern) was carried out retaining 60 first PCs (75% of variance conserved) of PCA and two discriminant eigenvalues. Separation between the three temporal clusters is visualized in the DAPC scatterplot with respect to the two first LD (Figure 6), especially for the heirloom cluster, which is the most distant one from to the others. No potato accession belonging to the heirloom cluster is found in the two more recent ones. Conversely, three potato accessions (among them *Gaumaise*) from the modern cluster are found in the heirloom cluster. Again, this is no surprise as, e.g., modern variety *Gaumaise* was created in 1955 from a self-fertilization of *Rosa* (syn. *Plate de Forenville*), a French heirloom. As expected, more potato accessions from the old cluster are found in the heirloom cluster. The higher proximity between the old and modern clusters explains why that many varieties from one cluster can be found in the other.

#### 3.3.4. Germplasm of 50-Year Breeding Groups

**Figure 7:**
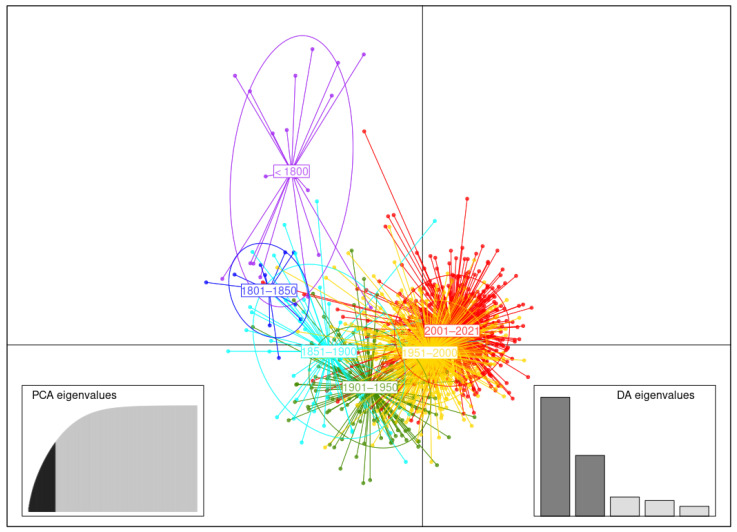
Discriminant analysis of principal components (DAPC) for the germplasm of 1219 potato accessions of 50-year breeding groups. The axes correspond to the first two linear discriminants (LD). Each ellipse represents a 50-year breeding cluster ([<1800], [1801–1850], [1851–1900], [1901–1950], [1951–2000], [2000–2021]), and each point represents an individual.

DAPC analysis using germplasm of 50-year breeding groups was carried out retaining 60 first PCs (65% of variance conserved) of PCA and five discriminant eigenvalues. The clusters comprising potato accessions belonging to 50-year breeding groups appear to be distributed in accordance with the chronology of the periods in the DAPC scatterplot with respect to the two first LD (Figure 7). Clusters from the early period (i.e., [<1800] and [1801–1850]) are roughly at the same level with respect to LD 1, while clusters following this period extend along the LD 1 axis. It should be noted that the latter we go in the timeline, the closer the clusters are, specifically for the two last 50-year breeding clusters. Thus, the first two temporal clusters (i.e., [<1800] and [1801–1850]) showed few potato accessions out of their predicted cluster, whereas the following chronological clusters overlap gradually over time.

### 3.4. AMOVA Analysis

The four previous models of genetic structuring were examined using AMOVA analyses to assess the genetic variability into within and among-group variation components. Results are summarized in Table 3. The proportion of the total SSR variation which resided among potato accessions released over different continents was 7.6%, while that explained by country origin was 3.2%. The proportion of the total SSR variation which resided among three temporal groups (4.4%) was higher than that which was found among accessions released over the four 50-year breeding periods (3.0%). Thus, the genetic variability among groups is proportionally higher when considering the spatial model of genetic structuring as compared with the temporal model.

## 4. Discussion

In a changing world, an environmentally sustainable increase in the productivity and nutritional value of crops can play a crucial role in ensuring global food security. Furthermore, human societies will remain vulnerable to the tragedies encountered in the past, for example, great famines caused by late blight. Additionally, climate change is predicted to have dramatic consequences for crops, including changes in geographical distribution, phenology, crop-pest synchrony, and increased risk of attacks by invasive pest species. In this study, three interconnected aspects of the spatiotemporal evolution of cultivated potato genetic diversity were examined to provide insights into its past and current genetic resources, with the aim of promoting appropriate strategies for breeding programs in line with the challenges of sustainable crop production and with regards to available genetic resources, either in the field or conserved in collections.

### 4.1. How Many Historical Introductions Are at the Origin of Modern Potato Lineages?

According to historical records, the germplasm of the modern cultivated potato is based on only a small number of introductions [25]. It has long been believed that two initial introductions occurred in the 16th century, the first into Spain around 1570 and a second one into England around 1586, with no evidence of further introductions having taken place up to the 19th century [15,64]. However, later discovered notarial documents record distinct shipments of tubers from the Canary Islands to Belgium in 1567 and to France in 1574 [65], consequently opening the door to further speculation as to the history of potato introduction and its multiple origins. Our SOM results reveal the occurrence of at least two distinct, but closely related, early lineages that have been maintained until today. One comprises (in neurons 10, 19, 28) the following heirloom potatoes (<1850): *Native of the Canary Islands*, *Makah Ozette*, *Yam*, *Raudar Islenskar*, *Lumpers*, *Raeburn’s Gregor cups*, *Rocks*, *Skerry Blue*, *Fransen*, *Pink Fir Apple*, and *Rosa*. A second one (neuron 2) comprises *Myatt Ashleaf*, *Highland Burgundy Red*, *Blanchard*, *Lagad Glas*, *Quarantaine de la Halle* and *Ratte*. Our results suggest that these two early branches have been maintained until today since the genetic groups to which they belong also include modern varieties which are closely related to them. In addition, no descendants of the Andigena potatoes were found in modern potatoes, while several were from the native potato accessions from Chiloe Island. Although the first identified lineages appeared to be closer to the accessions from Chiloe Island (neuron 22) and the second to Andigena accessions (neuron 5, 6), it remains difficult to connect these two lineages to strains historically recorded, the originally introduced ancestors having most likely disappeared.

A logical extension of this study would be to identify the source of these detected early lineages, which could be of upland “Andean” or/and lowland “Chilean” origin. Indeed, these two genetic groups result from an adaptation process targeting distinct loci [17,21]. Despite controversial hypotheses about the complex origin of the potato in Europe and its subsequent evolution [14,15,16,17,18,19], recent consensus based on molecular data makes clear that the earliest introductions of the potato came from both the Andes and Chile, so potatoes predominating in the 1700s would be descended from ancestors of Andean landraces (Andigenum group), while more recent ones would instead be descended from Chilean potatoes (Chilotanum group) which became predominant long before late blight epidemics [17,18,19,20]. According to previous studies [66,67,68,69], the following primitive cultivars *Lumpers*, *Myatts Ashleaf*, *Pink Fir Apple*, *Ratte*, *Fransen* have A-type ctDNA, which is typical of Andean potatoes, whereas *Yam*, released before late blight epidemics, has T-type ctDNA, typical of Chilean potato, as the vast majority of existing modern potato cultivars. However, since our SOM clustering has shown that *Yam* and *Lumpers* are grouped in neighboring clusters 10 and 19, the relationship between any resulting SOM grouping and the available ctDNA-type information cannot be established de facto. Consequently, it is at this stage nearly impossible to establish the source, Andean or Chilean, of these two identified lineages. To unravel this issue, it would be of interest to further differentiate these two identified earlier groups by identifying the cytoplasmic type for each accession concerned, for instance using the method of Hossaka and Sanetomo (2012) [70], which make it possible to distinguish up to six cytoplasm types.

From the 1850s, SOM temporal analysis has revealed the appearance of new genetic clusters which were relatively distant from the first lineages detected in the grid. This corroborates theories about reintroduction events from the US after the Great Famine in Europe in the mid-1800s. The genetic dissimilarity between these clusters is easily explained. In North America, potatoes were most likely introduced from Europe at the end of the 17th century. Several important introductions have since added to the original genetic material. For example, the Chilean clone *Rough Purple Chili*, introduced by C. Goodrich in the 1800s from Chile, appears in the pedigree of numerous modern varieties, and subsequent selection led to it becoming a founder of a line of descent which includes *Early Rose* [64]. *Early Rose* is itself an ancestor of a substantial proportion of American variety pedigrees spanning a century and a half of breeding work. *Early Rose* and its offspring were then used widely in the production of European varieties after the Great Famine [71]. The occurrence of recurring ancestors in the American and European countries, as well as in all other countries’ potato heritage, explains the overall proximity of the points in the spatial DAPC plots (Figure 4 and Figure 5).

From the 1900s until today, a reinforcement of preexisting clusters is found with numerous new accessions also appearing around preexisting clusters. Interestingly, the SOM network, especially in relation to the 1950s onwards, begins to further expand by occupying more adjacent neurons. This illustrates more intense activity in potato selection, by the means of increased exchange of varieties between countries and the enhancement of potato germplasm using exotic material as well as by targeting specific market niches [28,34,59]. Spatial DAPC analysis supports this finding by showing an identical progressive growth of both closeness and the creation of new varieties.

### 4.2. Are There Signs of Genetic Erosion over Time and Space in the Cultivated Potato?

Throughout its history, the cultivated potato has experienced severe and repeated selection pressures, resulting in some sequential putative genetic bottleneck events [24]. In particular, late blight pandemics and seed-borne viruses carried in tubers led together to a dramatic loss of many potato varieties across the globe in the 19th century [25]. Nevertheless, recent studies have shown that genetic erosion is almost absent in the potato [28]. Despite the accessible gene pool for conventional potato breeding and significant breeding efforts that have since been undertaken to avoid such further genetic losses, there are still concerns about both the narrowing genetic base of the modern potato germplasm and the limited genetic differentiation between potato subpopulations [29,39]. This would be due to (1) the small number of parental lines used for potato breeding as a result of the small number of introduction events that provided the genetic base of modern germplasm [25,64]; (2) the use of major contributing ancestors (MCAs) for a large proportion of the germplasms that make up prominent cultivars [29], especially since some MCAs have appeared numerous times in the pedigree of the cultivars, further accentuating the effect of genetic narrowing; and (3) the few introgressions of new exotic germplasm [27,35], as discussed below. However, our results demonstrate on the contrary that the genetic base seems to have widened increasingly over time and space.

From a temporal point of view, multivariate analyses show that the diversity evolved gradually in a structured way throughout the studied periods. This corroborates the work of Vos et al. (2017) [72], which showed differences in the genetic structure of temporal subsets of varieties. Even if genetic differentiation between temporal groups is perceptible, most SOM clusters include varieties belonging to all the breeding periods. Similarly, the distribution of DAPC temporal clusters encompasses most of the diversity found in earlier decades, especially concerning more recent periods. These results are explained by the recurrent use of old varieties in more recent crosses. The analysis of the pedigree information concerning these cases supports this conclusion. At allelic level, we note that the percentage of SSR alleles present in the old groups studied, but not detected in the more recent groups, was low. This allelic loss of ancient alleles is, however, largely compensated by the appearance of new alleles over time. Likewise, the allelic frequencies do not change significantly over time. This allelic stability is certainly facilitated by the highly heterozygous polyploid structure and the vegetative propagation mode of the potato, which makes it less prone to allelic loss as compared to many other more homogeneous crops. Consequently, our data support the idea that no significant genetic narrowing events have occurred over the past three centuries. It should nevertheless be noted that the number of alleles per SSR, especially of rare alleles, of the accessions belonging to the Andigenum group (Andigena native accessions) present in our panel was always higher than in accessions of the modern Chilotanum group (including native accessions from Chiloe Island), suggesting a reduced genetic diversity in modern potato cultivars relative to landraces. This is consistent with the works of Spooner et al. 2005 [17] and Esnault et al. 2014 [43].

From a spatial point of view, a genetic structure is identified in which the potato accessions appear more distant between continents than between countries. Accessions originating from European countries tend to cluster together, except for those from the UK, which fall between those from European countries and those from the US. The genetic proximity between the latter groups can be explained by historical introduction events from the UK to the Americas in the 17th century, and vice versa following European late blight epidemics in the mid-1800s [19]. Accessions originating from China are intermediate between those of the North American continent and Germany. This reflects the exchange of material in different breeding programs during cooperation between these two countries. Nevertheless, the multivariate analyses show that diversity also overlaps throughout the studied areas. This illustrates the practice of varieties used as progenitors being exchanged between countries that underlies constant worldwide gene flow. While SOM analysis reveals that clustering is highly connected with pedigree, some MCAs, especially those used at an international level, deviate somewhat from this grouping. When MCAs such as *Agria* (NLD), *Daber* (GER), *Katahdin* (USA), *Flourball* (GBR), *Early Rose* (USA) and *Mira* (DEU) are crossed with contrasting material (with the variety chosen depending on the available genetic resources in the breeding country) the resultant hybrids are split into different clusters in the grid. This has been reported in a previous study [59]. Although the varieties studied here were obtained from major European breeders, some continents, especially North America, Asia, and Eastern Europe, are underrepresented. The variety panel may therefore not accurately reflect the global gene pool. However, according to the online pedigree database [22], most of the varieties that could be argued to be ‘missing’ from this analysis have close parental affiliations with the studied varieties and should therefore not unduly influence the identified spatial structure.

### 4.3. Is the Impact of the Introduction of Exotic Potato Germplasm into the Modern Cultivated Potato Noticeable?

As for many cultivated crops, the use of wild relatives or landraces by breeders as a source of adaptative potential for the improvement of existing germplasm can have a significant impact in the cultivated potato [33]. The process of incorporation (base broadening), which relies on the development of populations adapted from foreign stock, is well advanced and is beginning to emerge in commercial breeding stock [32]. Likewise, the process of introgression, which involves the backcrossing into adapted stocks of selected genes providing desired characteristics, has achieved many successes in breeding programs over more than a century [35]. Recent studies have shown that the genetic structure of the potato gene pool, although weak, is mainly explained by origin, breeding objectives, and market niches [7,9,28,34,59,73]. However, it appears that only a small fraction of wild potato germplasm has been evaluated for its potential to contribute to improved cultivars [35,74]. This is essentially due to practical difficulties inherent to hybridization barriers [75] and to breeding program strategies that have mostly focused on introgressions of disease-resistance genes from wild potatoes [33,76]. Additionally, the introduction of exotic genetic variations often creates breeding products with characteristics that put them outside of the targeted market niche, with the consequence that commercial varieties often emerge from a relatively small gene pool [77]. As regards the process of introgression, backcrossing procedures aim at eliminating most of the phenotypic contributions from wild varieties apart from the specific traits for which they were targeted, thus limiting the allelic richness that could be drawn on [37]. The introduction of negative traits, which is inherent to the process, including those such as late maturity and extensive stolon development, can also bring significant concerns [76]. Consequently, the contribution of exotic germplasm to broadening the genetic base of the cultivated potato has likely been small, with the major contribution therefore coming from introductions in previous centuries which gave rise to earlier potato varieties [25]. Nevertheless, a recent study based on understanding the origins of cultivated potatoes from historical genomes found that 20th century European potatoes were unlikely to be descended directly from their 19th century predecessors, but rather to have received gene flow from wild potato species [20]. Introgression breeding could thus lead to an increase in genetic variation [28], as also evidenced in the cultivated tomato [78].

Our results revealed that current diversity is explained both by inheritance from ancestral cultivars and by the introduction of additional gene pools whose effect on the genetic base is noticeable over time. Many genetic clusters have appeared over time within the cultivated potato population, especially from the 1950s onwards. For instance, newly detected clusters at the origin of major emerging lineages have been due to the use of:

(1) *S. Tuberosum* ssp. Andigena *CPC 1673* in many breeding programs, which confers resistance to *G. rostochiensis* pathotypes Ro1 and Ro4 [36,79] as well as to *G. pallida* pathotype Pa2 [80]. Many resistant tetraploid potato cultivars have *S. tuberosum* ssp. Andigena *CPC 1673* in their pedigree, including *Agria*, *Alcmaria*, *Amaryl*, *Amex*, *Aminca*, *Carrera*, *Cherie*, *Elkana*, *Mara*, *Prominent*, and *Saturna*;
(2) *Maris Piper* and *Ulster Glade*, cultivars that have been bred with a dominant gene for resistance to cyst nematode, derived from *S. tuberosum* ssp. Andigena;
(3) *Lenape*, which has in its second-generation ancestors a wild potato native to the Chaco region in South America, *S. chacoense*, selected to provide the qualities of resistance to certain diseases, including late blight;
(4) *BRA 9089*, whose parents are from (Chilote x Svitez) and landrace cross, in the basis of important lineages in China with *Mira* (syn. *Ora*) as well as in Germany with *Axilia*, *Leander*, *Hessenkrone*, and *Sitta*;
(5) Improved clones *VTN 62-033-03* and *AM 66-0042* using *S. vernei* and *S. demissum* for targeted resistance to certain diseases and high starch yield. These two ancestors are highly represented in the pedigree of modern varieties.

In addition to the priority targeting of disease resistance and yield, specific market niches have clearly been exploited in parallel, such as fresh market, French fries processing, crisp processing, and starch industry. Thus, the ancestors responsible for the new genetic stratifications identified in the structure of the studied gene pool almost all originate from recent breeding programs using exotic material, either from wild potato relatives or from landraces.

## 5. Conclusions

By examining spatiotemporal trends in the genetic diversity of cultivated potatoes, our study demonstrates that no loss of diversity in this crop over the past three centuries should be feared. On the contrary, the genetic analysis presented here shows encouraging signs of improvement, thanks to the sensible and significant work which has been carried out by potato breeders, especially over the past century. From a practical perspective, the data collected through this research could be used to guide breeders, who were partners in this study, in the design of appropriate selection strategies, for instance by anticipating, in full knowledge of the facts, possible crop losses resulting from changing environments, and using available genetic resources already adapted to similar constraints. This can be done by filling gaps in the genetic patterns identified in breeding stock germplasm and by proposing parent candidates whose membership in relevant genetic clusters has been clearly identified. Finally, it would be of interest to confirm our data using more abundant and automatized molecular markers, such as SNP arrays. This could circumvent the known limits of microsatellite markers such as homoplasy and the problems associated with determining allele dosage inherent to polyploid data.

## Figures and Tables

**Table 1:** Summary of potato accessions number registered in the study with respect to country of origin and date of release.

Country	No. of Accessions	50-Year Temporal Group					
		Heirloom		Old		Modern	
		<1800	1801–1850	1851–1900	1901–1950	1951–2000	2001–2021
Netherlands	382	/	/	3	25	171	183
France	262	/	5	9	9	123	116
Germany	201	/	/	4	40	83	73
UK	142	1	5	26	49	39	22
USA	68	3	/	14	12	36	3
Austria	35	/	/	1	/	14	20
China	25	/	/	/	/	2	23
Denmark	23	/	/	/	/	9	14
Belgium	12	/	/	/	/	11	1
Czechia	10	/	/	/	5	5	/
Andes	9	9	/	/	/	/	/
Poland	8	/	/	/	1	7	/
RD Congo	8	/	/	/	1	7	/
Chiloe Island	3	3	/	/	/	/	/
Hungary	5	/	/	/	1	1	3
Ireland	4	/	1	1	/	1	1
Russia	4	/	/	/	2	2	/
Canada	2	/	/	/	/	2	/
Japan	2	/	/	/	/	2	/
Sweden	2	/	/	/	1	1	/
Ukraine	2	/	/	/	/	2	/
Others *	10	3	/	1	/	3	3
Total	1219	19	11	59	146	522	462

* “Others” brings together 10 countries (i.e., Australia, Canary Islands, Finland, Island, Italy, Luxemburg, Norway, New Zealand, Spain, and Ukraine) counting 1 potato accession.

**Table 2:** Information about the 35 SSR markers used in this study, where the marker locus, linkage group (LG), multiplex set, fluorescent dye labeling, allele size range, number of total, rare, and unique alleles, mean alleles per accession, heterozygosity frequency (H1) and PIC are given based on a panel of 1219 potato accessions.

Marker Locus	LG	Multiplex Set	Dye Labeling	Alleles Size Range	No of Total Alleles	No of Rare Alleles	No of Unique Alleles	Mean Alleles per Accession	H1	PIC
STG0016 ^g^	I	2	Ned	118–157	12	7	0	2.81	0.985	0.777
STM1049 ^d^	I	2	Ned	178–199	9	4	1	1.89	0.728	0.653
STM2020 ^d^	I	4	Ned	137–160	14	7	2	2.87	0.965	0.824
STM5127 ^g^	I	2	Hex	236–271	12	4	2	2.75	0.969	0.793
STG0006 ^g^	II	2	Fam	141–159	7	3	0	1.23	0.217	0.346
STI0036 ^f^	II	1	Hex	111–146	13	5	0	2.93	0.974	0.834
STM1064 ^d^	II	1	Hex	182–194	9	2	1	2.04	0.799	0.654
STI0013 ^f^	III	2	Fam	247–308	10	5	1	2.58	0.963	0.747
STI0050 ^f^	III	5	Hex	149–167	7	2	0	2.62	0.971	0.745
STI0001 ^f^	IV	5	Hex	177–198	8	2	1	2.46	0.909	0.752
STI0012 ^f^	IV	1	Fam	163–195	10	3	0	2.87	0.979	0.812
STM5140 ^e^	IV	4	Ned	162–201	8	3	0	2.73	0.982	0.766
LEMALX ^d^	V	4	Fam	117–133	8	4	2	1.91	0.720	0.656
STG0021 ^g^	V	3	Hex	108–140	9	3	1	2.55	0.960	0.745
STPOAC58 ^e^	V	3	Ned	227–247	12	6	1	2.53	0.925	0.763
STI0004 ^f^	VI	1	Hex	63–104	15	7	1	2.50	0.916	0.775
STI0011 ^f^	VI	5	Hex	56–77	10	3	1	2.28	0.888	0.717
STI0021 ^f^	VI	4	Ned	82–106	9	2	0	2.82	0.984	0.783
STI0033 ^f^	VII	3	Ned	111–135	8	2	0	2.75	0.976	0.769
STM1052 ^d^	VII	1	Ned	196–262	14	5	2	2.62	0.934	0.816
STM3009 ^d^	VII	5	Fam	138–170	18	13	2	2.58	0.932	0.779
SSR1 ^b^	VIII	4	Fam	198–225	15	5	1	2.73	0.970	0.793
STGBSS ^c^	VIII	1	Ned	121–142	13	5	0	2.20	0.824	0.715
STM1104 ^d^	VIII	2	Hex	160–181	15	7	1	2.11	0.792	0.707
STWAX-2 ^a^	VIII	1	Fam	209–244	15	7	1	2.48	0.938	0.759
STI0002 ^f^	IX	5	Ned	99–132	18	11	5	2.42	0.903	0.765
STI0014 ^f^	IX	2	Hex	113–132	8	4	0	2.33	0.942	0.668
STM3012 ^d^	IX	3	Hex	136–207	11	4	2	2.20	0.825	0.712
STG0025 ^g^	X	3	Fam	193–205	7	3	0	1.91	0.847	0.546
STI0023 ^f^	X	5	Ned	149–217	22	17	6	2.40	0.936	0.718
STG0001 ^g^	XI	3	Fam	126–145	15	6	1	2.85	0.971	0.831
STM0037 ^d^	XI	5	Fam	63–101	16	10	2	2.64	0.931	0.810
STM2005 ^d^	XI	4	Hex	147–191	6	2	1	2.57	0.961	0.726
STI0030 ^f^	XII	3	Fam	84–105	11	3	1	2.73	0.980	0.777
STM1097 ^d^	XII	4	Hex	225–280	13	8	2	2.01	0.746	0.667
Sum					407	184	41			
Mean					11.6	5.3	1.2	2.45	0.893	0.734

The sources of the SSR markers are indicated in exponent with ^a^ Veilleux et al. (1995), ^b^ Kawchuk (1996), ^c^ Provan et al. (1996), ^d^ Milbourne et al. (1998), ^e^ Ghislain et al. (2004), ^f^ Feingold et al. (2005), and ^g^ Ghislain et al. (2009).

**Table 3:** Results of analyses of molecular variance (AMOVA) for the germplasm of 1219 potato accessions with four models of genetic structuring.

Model	*df*	Sum of Square	Variance Component	Percentage of Variation	*p*-Value ^a^
Germplasm of various continental origins					
*among continents*	6	797.63	2.71	7.6	<0.0001
*within continent*	1137	39,694.63	32.75	92.4	<0.0001
Germplasm of various country origins					
*among countries*	6	1194.31	1.06	3.2	<0.0001
*within countries*	1134	39,297.95	32.39	96.8	<0.0001
Germplasm of three temporal groups ^b^					
*among temporal groups*	2	653.31	1.51	4.4	<0.0001
*within temporal groups*	1140	39,838.95	32.76	95.6	<0.0001
Germplasm of 50-year groups					
*among breeding periods*	5	959.03	1.00	3.0	<0.0001
*within breeding periods*	1137	39,533.23	32.59	97.0	<0.0001

^a^ The probability that the among-group (or among-continent, among-country, among-temporal, among-50-years) variance component was larger than zero, as computed based on random permutations. ^b^ Germplasm temporal groups includes heirloom, old, and modern potato varieties.

## Data Availability

Datasets supporting the findings of this study are available from the corresponding author upon reasonable request.

## Supplementary Materials

The following supporting information can be downloaded at: https://www.mdpi.com/article/10.3390/biology11040604/s1, Table S1: Accessions background information. Table S2: Data matrix of SSR alleles per variety. Table S3: Appearance and disappearance of SSR alleles over time (50-year series).
